# Cancer Metabolism and the Evasion of Apoptotic Cell Death

**DOI:** 10.3390/cancers11081144

**Published:** 2019-08-09

**Authors:** Aditi Sharma, Lawrence H. Boise, Mala Shanmugam

**Affiliations:** Department of Hematology and Medical Oncology, Winship Cancer Institute, School of Medicine, Emory University, Atlanta, GA 30322, USA

**Keywords:** apoptosis, BCL-2, metabolism

## Abstract

Cellular growth and proliferation depend upon the acquisition and synthesis of specific metabolites. These metabolites fuel the bioenergy, biosynthesis, and redox potential required for duplication of cellular biomass. Multicellular organisms maintain tissue homeostasis by balancing signals promoting proliferation and removal of cells via apoptosis. While apoptosis is in itself an energy dependent process activated by intrinsic and extrinsic signals, whether specific nutrient acquisition (elevated or suppressed) and their metabolism regulates apoptosis is less well investigated. Normal cellular metabolism is regulated by lineage specific intrinsic features and microenvironment driven extrinsic features. In the context of cancer, genetic abnormalities, unconventional microenvironments and/or therapy engage constitutive pro-survival signaling to re-program and rewire metabolism to maintain survival, growth, and proliferation. It thus becomes particularly relevant to understand whether altered nutrient acquisition and metabolism in cancer can also contribute to the evasion of apoptosis and consequently therapy resistance. Our review attempts to dissect a causal relationship between two cancer hallmarks, i.e., deregulated cellular energetics and the evasion of programmed cell death with primary focus on the intrinsic pathway of apoptosis.

## 1. Introduction

Cancer cells engage various mechanisms to evade apoptosis. The intrinsic pathway of apoptosis has been shown to be frequently disrupted in cancer cells and is closely regulated by cellular metabolism. While oncogenes and tumor suppressors reprogram tumor metabolism, microenvironment or therapy-imposed stresses can additionally rewire metabolism, creating new metabolic dependencies. Direct evidence that metabolic changes promote resistance to therapy via alterations of BCL-2 family expression is sparse. Metabolites like glucose and glutamine however have been shown to regulate the expression and binding properties of specific BCL-2 proteins. The strongest evidence that cancer metabolism regulates programmed cell death to promote resistance comes indirectly from numerous studies showing that targeting metabolism alters the apoptotic threshold consequently sensitizing resistant cancer to various therapies. There is opportunity for developing therapies that co-target metabolic vulnerabilities and the BCL-2 family of proteins. We thus review the intrinsic pathway of apoptosis and its close relationship with metabolism.

## 2. Apoptosis: Extrinsic and Intrinsic Programmed Cell Death

Apoptosis is a mode of programmed cell death essential for maintaining tissue homeostasis by elimination of unwanted, superfluous, and damaged cells [1]. Apoptosis occurs discretely in individual cells of our body and is a highly regulated energy dependent process. Deregulation of apoptosis is involved in the pathogenesis of several diseases like neurodegenerative conditions, which involve excessive apoptosis, as well as cancer, which, in contrast, is characterized by accumulation of cells exhibiting insufficient engagement of the apoptotic machinery and evasion of apoptosis [2,3,4]. There are two primary pathways that lead to apoptosis; the intrinsic pathway of apoptosis and the extrinsic pathway of apoptosis. Both pathways result in the activation of cysteine aspartyl-specific proteases or ‘caspases’, which are the final effectors of apoptosis and cleave several proteins leading to cell death [5,6,7]. The extrinsic pathway can be engaged by activation of death receptors of the tumor necrosis factor (TNF) superfamily such as Fas (Apo/CD95), TNF Receptor 1 (TNFR1), TNF-related apoptosis-inducing ligand (TRAIL) receptors, etc., located on the cell surface, by binding with their specific ligands [8,9]. On the other hand, induction of the intrinsic (mitochondrial) pathway is primarily regulated by the B cell lymphoma (BCL-2) family of proteins [10] and is activated by internal stress sensors in response to cellular stresses like nutrient deprivation, DNA damage, hypoxia, etc. Detachment of cells from the extracellular matrix can also induce a form of apoptotic cell death called ‘anoikis’, which acts to control the growth and re-attachment of detached cells to a different matrix [11]. Resistance to anoikis is an attribute of cancer cells with metastatic potential [12,13]. Anoikis can engage both extrinsic as well as the intrinsic pathway of apoptosis [13,14].

Apoptosis is biochemically and morphologically distinct from other forms of programmed cell death implicated in cancer such as ferroptosis [15,16]. Ferroptosis was originally discovered as an iron-dependent form of oxidative cell death induced by inhibition of cystine uptake by the cystine/glutamate antiporter (xCT) using small molecule inhibitors, such as Erastin or excess glutamate [15]. Ferroptosis is initiated by a decrease or inhibition of glutathione peroxidase 4 (GPX4) activity. GPX4 catalyzes the reduction of lipid peroxides while oxidizing glutathione (GSH), a key player in the cellular antioxidant defense. Depletion of GSH by inhibition of xCT, for instance, or direct inhibition of GPX4 results in accumulation of lipid peroxides, which are converted to lipid ROS by the action of reactive iron cations such as Fe^2+^ resulting in ferroptotic cell death. In another study, glutaminolysis, fueled by glutamine along with iron carrier, transferrin, were found to be critical regulators of ferroptosis [17]. Hence, ferroptosis is linked to dysregulation of iron and ROS metabolism and lipid peroxidation pathways and is closely linked to glutamine metabolism. While, ferroptosis warrants a mention here, this review will primarily focus on the intrinsic apoptotic pathway of cell death and its relationship with cancer metabolism.

The key regulators of the intrinsic pathway of apoptosis, are the BCL-2 family of proteins that are broadly classified as either anti-apoptotic or pro-apoptotic. BCL-2 family members are characterized by containing at least one of four BCL-2 homology (BH1-4) domains. Induction of the intrinsic pathway of apoptosis is initiated by the release of pro-apoptotic BH3-only ‘activators’ (BCL-2 interacting mediator of cell death (BIM), P53-upregulated modulator of apoptosis (PUMA), or truncated BH3 interacting domain death agonist (tBID)) from anti-apoptotic multi-domain BCL-2 family members (BCL-2, BCL-x_L_, myeloid cell leukemia 1 (MCL-1), BCL-w and A1) to activate multi-domain ‘effectors’, BCL-2 associated X protein (BAX) and BCL-2 antagonist/killer (BAK) [18,19].

**Figure 1:**
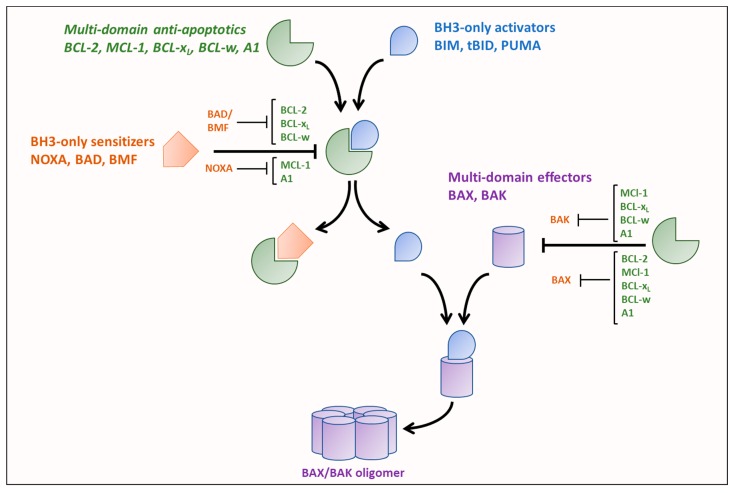
Unified model of BCL-2 protein family interactions. BH3-only activators (BIM, tBID, and PUMA) can bind to the multi-domain anti-apoptotics (BCL-2, MCL-2, BCL-x_L_, BCL-w, and A1) as well as the multi-domain pro-apoptotic effectors (BAX and BAK), whereas each of the BH3-only sensitizers (NOXA, BAD, and BMF) can only bind to a subset of the multi-domain anti-apoptotics. Binding of BH3-only activator to BAX/BAK promotes the oligomerization of BAX/BAK. BAX and BAK can be inhibited by a subset of the anti-apoptotics.

The activation of the intrinsic pathway of apoptosis is regulated by a delicate balance of pro- and anti-apoptotic BCL-2 proteins. BH3 domain-only proteins have different binding affinities for the multi-domain pro- and anti-apoptotic proteins dictated by differences in the amino acid sequences of their BH3 domains [20,21]. Additionally, the BH3 domain-only activator proteins bind to all multi-domain anti-apoptotics with high affinities while the BH-3 domain-only sensitizers have selective binding affinities for the anti-apoptotics, as shown in Figure 1. Furthermore, activators also have variable binding affinities for the effectors, for example, BIM has been shown to preferentially activate BAX while BID preferentially activates BAK [22]. BH3-only activators are released either by reduction in expression of an anti-apoptotic to which they are bound or displacement from the anti-apoptotic by a BH3-only sensitizer (such as NOXA, BCL-x_L_/BCL-2 associated death promoter (BAD), and BCL-2 modifying factor (BMF)). The released BH3-only activators can then activate BAX and BAK, promoting their oligomerization and result in subsequent mitochondrial outer membrane permeabilization (MOMP), thus releasing proteins of the intermembrane space such as cytochrome c, second mitochondria-derived activator of caspase (SMAC)/DIABLO, endonuclease G, and OMI into the cytosol [23] to activate distinct steps of the apoptotic cascade. Besides differences in binding tendencies, expression levels and post-translational modifications of the different BCL-2 family proteins also play a major role in determining if apoptosis is initiated [21,24,25,26].

The extrinsic pathway can interact with the intrinsic pathway through BID which is cleaved by caspase-8 to its truncated active form, tBID [27]. This cross-talk between the extrinsic and intrinsic pathways of apoptosis is crucial in cells that are unable to initiate apoptosis only by activation of death receptors and require additional engagement of the intrinsic pathway. This has important implications for approaches which use TRAIL and TRAIL receptor-based therapies to engage the extrinsic pathway as cancer cells which require tBID activation of intrinsic pathway may be rendered resistant by changes in the levels of BCL-2 proteins (such as overexpression of BCL-2) upon death receptor activation. [28]. Further, studies linking metabolism to activation of the extrinsic pathway of apoptosis by death receptor-ligand interactions are scarce and often require involvement of the intrinsic pathway [10,29,30,31,32]. Therefore, we have limited the scope of this review to the intrinsic pathway and its regulation by cancer metabolism.

## 3. Cancer and Deregulation of BCL-2 Family Proteins

Evasion of cell death is one of the hallmarks of cancer [33]. Cancer cells employ various means of preventing cell death including deregulation of the intrinsic apoptosis pathway. Upregulation of the anti-apoptotic BCL-2 proteins and loss of pro-apoptotic BH3 proteins has been observed in many cancers [34,35]. In fact, BCL-2 was identified in the t(14,18) chromosomal translocation in follicular lymphoma, which results in fusion of the BCL-2 gene locus with in the immunoglobulin heavy chain locus on chromosome 14 with reciprocal translocation of the variable heavy chain genes to chromosome 18 [36,37,38]. Upregulation of BCL-2 has also been observed in a number of other hematological malignancies as well as solid tumors [39]. Similarly, increased levels of BCL-x_L_ and MCL-1 have been detected via different mechanisms and shown to promote tumorigenesis [39,40,41]. Many cancers engage mechanisms for stabilization of MCL-1, which is a highly unstable protein that exhibits rapid turnover due to proteasomal degradation [42,43,44]. On the other, hand downregulation of pro-apoptotic proteins is another means of avoiding apoptosis initiation. Loss of p53 tumor suppressor, which upregulates pro-apoptotic BH3-only proteins such as PUMA, BID, and NOXA, has been observed in various cancers [45,46,47,48]. Pro-apoptotic effectors—BAX and BAK—have also been found to be downregulated in multiple cancers [49,50,51].

However, despite of successful evasion of apoptosis, cancer cells are generally more susceptible to apoptosis than normal cells. The genetic complexity, microenvironment (hypoxia, nutrient deprivation/competition, and pH), and therapy-related stress bring cells more proximal to the apoptotic threshold of cancer cells. Cancer cells thus often express elevated levels of pro-apoptotic BH3-only proteins. Concordantly, to sequester unbound pro-apoptotics and thereby prevent MOMP, cancer cells generally upregulate anti-apoptotic BCL-2 family proteins and, as a result, contain higher levels of pro-apoptotic activators bound to anti-apoptotic proteins. Such cells exhibiting elevated levels of anti-apoptotics bound to pro-apoptotics and proximal to the apoptotic threshold are considered to be ‘primed’ for apoptosis [52].

## 4. Cancer Metabolism

Cancer cells exhibit altered nutrient acquisition and metabolism to sustain biosynthetic, bioenergetic and redox homeostasis demands. Alterations in specific metabolites also have implications on gene expression, protein expression and/function by, for example, epigenetic and posttranslational modifications. In particular, cancer cells exhibit elevated uptake of glucose and glutamine. Numerous cancer cell types exhibit elevated expression of the glucose transporter GLUT1 allowing for facilitative glucose uptake [53,54]. GLUT1 is a high affinity glucose transporter and proximal rate-limiting step in glucose metabolism [54,55,56]. It is the most frequently implicated GLUT family member in cancer and linked to poor survival and prognosis [57,58]. Glycolysis is the first major pathway involved in glucose catabolism enabling the retention of glucose within the cell when hexokinase phosphorylates glucose to glucose-6-phosphate. The ensuing steps of glycolysis then allow for the anaerobic synthesis of NADH, ATP, and pyruvate. Glucose-6-phosphate is also channeled into the pentose phosphate pathway (PPP) for synthesis of nucleotide precursors and production of nicotinamide adenine dinucleotide phosphate (NADPH), which is required for maintaining redox homeostasis as well as for reductive biosynthesis of fatty acids [59,60,61]. The irreversible oxidative arm of the PPP contributes to cellular NADPH pools while the reversible non-oxidative arm produces nucleotide precursor ribose-5-phosphate and glycolysis intermediates such as fructose-6-phosphate and glyceraldehyde-3-phosphate. Cancer cells utilize PPP to support cell growth and proliferation and for antioxidant defense via NADPH synthesis. Depending upon cellular requirements for NAPDH synthesis and/or ribose-5-phosphate, the oxidative and/or the nonoxidative arms of the PPP may be activated. The PPP was also identified as a prosurvival pathway in AML cells with high mammalian target of rapamycin complex 1 (mTORC1) activity and inhibition of glucose-6-phosphate dehydrogenase (G6PD), the first rate-limiting enzyme of the oxidative arm of the PPP, selectively targeted AML cells [62]. Further, mTORC1 has been shown to circumvent glycolysis inhibition via the oxidative PPP in a number of different cancers [63]. Pyruvate is further transported into the mitochondria and converted to acetyl-CoA, which feeds into the tricarboxylic acid (TCA) cycle producing various TCA cycle intermediates and NADH (reduced nicotinamide adenine dinucleotide) and FADH_2_ (flavin adenine dinucleotide). NADH and FADH_2_ fuel oxidative phosphorylation (OXPHOS) to generate adenosine triphosphate (ATP). Even though generation of lactate from glucose is less energy efficient cancer cells primarily convert glucose to lactate regardless of the oxygen concentration. This phenomenon of aerobic glycolysis is termed the ‘Warburg effect’ [64,65,66]. The inherent reliance of cancer cells on aerobic glycolysis despite the presence of functional mitochondria and clear requirement for mitochondrial metabolism [67,68,69] in tumor development suggests the unique biology sustained by glucose metabolized in the glycolysis/PPP vs. mitochondrial pathways.

Several studies have also shown that cancer cells are also highly dependent on glutamine as a source of carbon and nitrogen for the synthesis of nucleotides, amino acids including glutamate and aspartate, and hexosamines, as well as for anaplerotic replenishment of TCA cycle intermediates and OXPHOS [70,71,72,73]. Importantly, glutamine is metabolized to glutamate—an amino acid that is critical in the synthesis of GSH—and thus, glutamine is also important for redox homeostasis in cancer cells [74,75,76]. While low levels of ROS have been shown to upregulate pathways promoting proliferation and as well as adaptive stress responses promoting tumorigenesis, higher ROS can upregulate cell death pathways [77,78,79]. Due to their high metabolic activity, cancer cells are faced with higher levels of endogenous reactive oxygen species (ROS) [79,80,81,82]. This necessitates synthesis of antioxidants such as GSH in order to maintain redox homeostasis and cancer cells may, thus, upregulate the import of nutrients and metabolic enzymes involved in the antioxidant defense.

Activation of oncogenes such as RAS, AKT, and MYC as well as loss of tumor suppressor genes such as p53 drive aerobic glycolysis in cancer cells [83]. RAS promotes glycolysis through activation of mTOR and upregulation of hypoxia inducible factors (HIFs) [84,85,86]. HIF1α in turn upregulates several genes promoting glycolysis as well as glucose transporters (GLUT1 and GLUT3) [87,88]. Elstrom et al., 2004, demonstrated that activation of AKT and the ensuing increase in aerobic glycolysis was sufficient to maintain survival and leukemogenesis of growth factor-deprived FL5.12 cells [89]. Under normal conditions p53 inhibits glycolysis through downregulation of glucose transporters (GLUT1 and GLUT4) and hexokinase 2 (HK2) enzyme and inhibition of HIF1α [90,91,92]. p53 also upregulates TP53-induced glycolysis and apoptosis regulator (TIGAR), which indirectly inhibits phosphofructokinase 1 (PFK1), another important glycolysis enzyme, thereby channeling glucose-6-phosphate into the PPP [93]. Therefore, loss of p53 promotes a glycolytic phenotype. Yun et al., 2009, showed that colorectal cell lines with mutant KRAS or BRAF upregulated GLUT1 and exhibited higher glucose uptake and glycolysis, which could be targeted with an inhibitor of hexokinase, 3-bromopyruvate (3-BrPA) [94]. They further demonstrated that the cell lines with the mutant alleles had a growth advantage in low glucose conditions and low glucose conditions selected for clones with KRAS or BRAF mutations. Moreover, the upregulation of GLUT1 was found to be independent of HIF1α suggesting that low glucose conditions may select for KRAS or BRAF mutations while hypoxia may favor PI3K, c-MYC, or TP53 mutations [94]. MYC, on the other hand, upregulates glucose and glutamine metabolism independent of the PI3K/AKT pathway [95,96]. Cells with oncogenic KRAS mutations or MYC upregulation have been shown to be dependent on glutamine for synthesis of amino acids, nucleotides and GSH [97,98,99]. MYC upregulation was associated with induction of glutaminase (GLS1), glutamate dehydrogenase (GDH) and the glutamine transporter, ASCT2/SLC1A5 [96,100,101,102,103]. Thus, oncogenes and tumor suppressors reprogram tumor metabolism and microenvironment-driven nutrient deprivation/hypoxia can additionally rewire metabolism creating new metabolic dependencies.

## 5. Intersection of Cancer Metabolism and BCL-2 Proteins

Many members of the BCL-2 family have been shown to be under metabolic control and are regulated by nutrient deprivation stresses. While an elevation in β-oxidation, ATP synthesis, and redox balance have implications in anoikis resistance [104] and glutamine metabolism in ferroptosis [17], we will focus on studies highlighting metabolic regulation of the BCL-2 protein family in this section. The highly labile BCL-2 protein MCL-1 has been reported to be stabilized by high glucose metabolism through different mechanisms. Zhao et al., 2007, showed that MCL-1 levels are regulated by Glycogen Synthase Kinase 3α and 3β (GSK-3α/3β), which phosphorylates MCL-1 at serine-159 and targets it for ubiquitination and degradation. Increased glucose metabolism increases phosphorylation of GSK-3α/3β at serine 21 and 9 by protein kinase C, negatively regulating its activity, thereby, preventing phosphorylation of MCL-1 and its subsequent degradation [105]. Pradelli et al., 2009, showed that reduction in MCL-1 levels upon glucose deprivation is regulated by inhibition of its translation through AMP-activated protein Kinase (AMPK) activation and mTOR inhibition [106]. Further, NOXA has been shown to induce apoptosis under glucose deprivation or by glycolysis inhibition by glucose analog 2-deoxy glucose (2-DG) with concordant decline in MCL-1 levels in different cancers [107,108,109,110]. Additionally, glucose limitation induces apoptosis through the ERK2/eIF-2α/ATF4-dependent pathway and upregulation of BID which could be reversed by supplementation with glutamate and α-ketoglutarate [111].

Glucose deprivation has also been shown to induce BH3-only sensitizer PUMA via induction of p53 which regulates the transcription of PUMA [112]. Moreover, Coloff et al., 2010, showed that PUMA and BIM are both induced upon glucose deprivation but only PUMA expression level is suppressed again upon restoring mitochondrial metabolism by supplementation with methyl pyruvate in the absence of glucose [113]. Further, they also showed that AKT promotes glycolysis and is required to reduce PUMA levels and apoptosis upon interleukin-3 (IL-3) withdrawal. Another mechanism through which AKT may prevent apoptosis is by increasing the binding of hexokinase to the mitochondrial membrane, thereby promoting its interaction with the outer membrane voltage-dependent anion channel (VDAC), and, in turn, preventing VDAC closure and mitochondrial membrane hyperpolarization that precedes cytochrome c release [114]. Further investigation of the mechanism by which AKT inhibited apoptosis led to the finding that AKT inhibited tBID mediated BAX and BAK oligomerization on growth factor withdrawal by promoting association of hexokinase with the mitochondrial membrane. Glucose deprivation was found to attenuate AKT induced inhibition of tBID mediated apoptosis by promoting dissociation of hexokinase from the mitochondrial membrane. Supporting these observations, overexpression of tBID also promoted dissociation of hexokinase from the mitochondrial membrane which was attenuated by activated AKT. Moreover, tBID mediated apoptosis was also inhibited by ectopic expression of the catalytically active amino-terminal region of hexokinase II containing its mitochondria-binding domain, further confirming the direct involvement of hexokinase in apoptosis [115].

Interestingly, glucose deprivation and treatment with 2-DG were found to result in different cell fates in mantle cell lymphoma. 2-DG resulted in cell death by downregulation of MCL-1 via the AMPK/mTOR pathway, while on the other hand, glucose deprivation inhibited cell death and maintained expression levels of BCL-2, BCL-x_L_, as well as MCL-1 by activation of the AKT and the subsequent inactivating phosphorylation of GSK-3β [116]. 2-DG has been shown to enhance ABT-737-induced apoptosis in lymphoma cells via upregulation of BIM that was reversed by supplementation with mannose [117]. Further, CHOP was found to be induced suggesting that apoptosis was initiated via the UPR-CHOP-BIM axis. Oxidative stress by co-treatment with 2-DG and 6-aminonicotinamide, which inhibits the PPP was also found to result in mitochondrial dysfunction and stimulate the intrinsic pathway of apoptosis in malignant cells [118].

Besides glucose deprivation other nutrient stresses such as amino acid deprivation can affect apoptosis. For example, both amino acid and glucose deprivation can activate the GCN2 (general control non-derepressible 2) kinase that phosphorylates eIF2α which in turn upregulates ATF4 translation. Glutamine depletion was found to induce cell death via upregulation of PUMA and NOXA in MYCN-amplified neuroblastoma [111]. This increase in PUMA and NOXA was mediated by ATF4 induction via the GCN2-eIF2a pathway. Further, nutrient deprivation can also result in the shift in the binding of pro-apoptotics to anti-apoptotics along with inducing changes in the expression levels. We have shown that glutamine deprivation results in upregulation of BIM and increased binding of BIM to BCL-2 in multiple myeloma (MM) [119]. Treatment of glutamine-deprived MM or 6-diazo-5-oxo-L-norleucine (DON)-treated MM patient samples and MM cell lines with the BCL-2 antagonist venetoclax (ABT-199) resulted in increased apoptosis. The glutamine-deprivation induced sensitization to venetoclax was partially reversed with α-ketoglutarate supplementation supporting the role of glutamine metabolism in regulating BIM induction and elevation of BCL-2 dependence in MM cells.

Recently a link between the electron transport chain (ETC) activity and the BCL-2 proteins has emerged. Chan et al., 2015, identified BCL-2 in a large-scale RNA interference (RNAi) screen to be synthetically lethal to Isocitrate Dehydrogenase 1 (IDH1) mutant expressing Acute Myeloid Leukemia (AML) and found that venetoclax selectively targeted mutant IDH1/2 AML [120]. This dependence of mutant AML on BCL-2 was found to be mediated by accumulation of oncometabolite (R)-2-hydroxyglutarate, which in turn inhibited Complex IV of the electron transport chain. Moreover, treatment of mutant IDH1/2 AML cell lines with cell permeable precursor of (R)-2-hydroxyglutarate, octyl-(R)-2-hydroxyglutrate sensitized the cells to venetoclax (ABT-199) [120]. In another study, a loss-of-function CRISPR/Cas9 knockout screen in AML identified heme biosynthesis in regulating apoptosis and sensitivity to venetoclax. Disruption of heme biosynthesis was found to deplete the ETC Complexes II-IV with a relatively higher effect on Complex IV than Complexes II and III. Treatment with succinyl acetone (SA) was found to attenuate the activity of Complexes II-IV as well as synergize with venetoclax induced death [121]. The authors further proposed that depolarization of the mitochondrial membrane due to heme depletion affected the integrity of the mitochondrial membrane and potentiated MOMP and apoptosis. Recent work by Chen et al., 2019, further demonstrated that disruption of mitochondrial function and dynamics sensitizes AML to venetoclax [122]. In this work, a CRISPR/Cas9 knockout screen was used to identify genes whose deletion synergized with venetoclax treatment. Several genes involved in mitochondrial structure and function were identified. One of the top-scoring candidates CLPB, which encodes a mitochondrial AAA+ ATPase chaperonin was also found to be upregulated in AML patients. Depletion of CLPB was shown to impact mitochondrial structure, induce mitochondrial stress response via upregulation ATF4 and its downstream targets as well as sensitize cells to venetoclax [122]. Our recent observations (under review) demonstrate that the succinate ubiquinone reductase activity of Complex II of the ETC is a predictor and target for venetoclax sensitization in MM [123].

Several studies have also demonstrated cross-talk between apoptosis and autophagy. Cancer cells frequently encounter nutrient deprivation stress and autophagy can promote their survival by recycling biomolecules via degradation of endogenous cellular components. Autophagy has also been shown to prevent cell death by apoptosis by degrading apoptosis regulators (e.g., degradation of caspase-8 in some TRAIL-induced apoptotic cells) [124,125]. On the other hand, autophagy can promote cell death through the engagement of cell death pathways [126,127]. Additionally, anti-apoptotic BCL-2 family proteins (BCL-2, MCL-1, and BCL-x_L_) can to bind BH3 domain only autophagy regulator Beclin-1 [128], which plays a key role in the initiation of autophagy [129]. Binding of these BCL-2 proteins to Beclin-1 inhibits the formation of pre-autophagosomal structure and prevents autophagy from occurring. Downregulation of MCL-1 due to nutrient deprivation as well as deletion of MCL-1 in cortical neurons can attenuate its inhibitory effect on autophagy or apoptosis and either pathway can ensue depending upon expression of Beclin-1 or BAX [130]. Further, Beclin-1 can be displaced from the anti-apoptotic BCL-2 proteins by BH3 only proteins such as BID, PUMA, BAD, and NOXA [131,132]. Therefore, changes in the metabolic state can impact both apoptosis and pro-survival/pro-apoptotic autophagy and their cross-talk.

BCL-2 family members also play a key role in regulating glucose and lipid metabolism. Phosphorylation of BH3-only BAD at Ser-155 was found to upregulate glucose oxidation by the mitochondria through interaction with glucokinase enzyme, which catalyzes the conversion of glucose to glucose-6-phosphate in hepatocytes [133]. While glucokinase has limited expression in other cells of the body, this observation has important implications as its isoform hexokinase II, is overexpressed in various cancers [134,135]. Further, glucose deprivation was found to reduce the phosphorylation on BAD and result in subsequent BAD-dependent apoptosis. The phosphorylated form of BAD has also been shown to play a role in glucose-dependent secretion of insulin by β cells [136]. Phosphorylation of BH3-only NOXA by cyclin-dependent kinase 5 (CDK5) has been shown to increase glucose flux into the pentose phosphate pathway [137]. Additionally, glucose deprivation and inhibition of CDK5 were shown to result in apoptosis through dephosphorylation of NOXA and restoration of its pro-apoptotic function. On the other hand, high glucose concentration can promote phosphorylation of NOXA at Ser-13 position. This phosphorylated form of NOXA binds MCL-1 but is unable to initiate MOMP and apoptosis. In addition, tBID has been reported to inhibit carnitine palmitoyl-transferase-1 (CPT1), thereby, preventing lipid transport and fatty acid (FA) oxidation in the mitochondria [138]. This tBID induced inhibition of FA oxidation was found to result in accumulation of FA metabolites such as palmitoyl-CoA. Palmitoyl-CoA can affect mitochondrial integrity and function and is also a precursor for synthesis of ceramide, which is an important mediator of programmed cell death [139,140,141]. Thus, this work highlights a close relation between the lipid metabolism and the apoptotic machinery implicating a direct involvement of lipid metabolites in the process of apoptosis.

BCL-2 proteins have been shown to regulate mitochondrial morphology and dynamics apart from their role in MOMP [142]. BAX and BAK were found to promote mitochondrial fusion in healthy cells [143,144]. Further, BAX and BAK are involved in the process of mitochondrial permeability transition pore (MPTP) formation in necrosis [145,146]. BCL-x_L_ has been shown to increase the rate of mitochondrial fission, fusion, and biomass in cultured neurons, as well as plays a neuroprotective role by promoting synapse formation [147,148]. In addition, BCL-2 proteins also play a role in regulation of metabolism at the inner mitochondrial membrane where they localize. BCL-2 was found to bind to cytochrome c oxidase and cyclophilin D and regulate mitochondrial respiration [149,150]. BCL-x_L_ has been shown to maintain mitochondrial membrane potential by directly modulating F_1_F_0_-ATP synthase and preventing proton leak into the mitochondrial matrix [151]. Further, MCL-1 deletion has been shown to result in a number of mitochondrial defects, suggesting an alternating role of MCL-1 in maintaining mitochondrial integrity and function [152].

In summary, there is considerable evidence that BCL-2 proteins are under metabolic regulation and in some instances can also modulate metabolism. Alterations in tumor metabolism can potentiate the ability of the cell to evade apoptosis. Therefore, the metabolic state has important implications for therapy as cancer cells can indirectly become resistant to chemotherapy by rewiring/reprogramming metabolism and, thus, changing their BCL-2 protein landscape.

## 6. Implications for Therapy

Most chemoresistance involves reduced drug sensitivity and impaired ability to execute apoptosis. The inability to effectively target BCL-2 proteins accounts for resistance to bortezomib [153,154,155], rapamycin [156], cyclin-dependent kinase inhibitors [157], ABT 737 [158], and death receptor (Fas/TRAIL)-induced apoptosis [106] in various cancers including MM. MM, acute myelogenous and lymphocytic leukemia, and various solid tumor cells and refractory cancers subject to prior chemotherapy, exhibit reduced “priming”, i.e., exhibit suboptimal quantities of BH3 activators bound to anti-apoptotics, thereby increasing the threshold level required to induce apoptosis [159,160,161,162]. Thus, developing strategies to increase the primed state could potentially circumvent resistance. Such approaches can potentially sensitize to existing therapy and also enhance sensitivity to BH3 mimetics/BCL-2 protein antagonists providing alternative routes to apoptosis induction.

The BH3 activator BIM is highly expressed in cells of hematopoietic origin [163], and evaluation of BH3 activators bound to the anti-apoptotic BCl-2 proteins in MM demonstrated that BIM is the most relevant BH3 activator dictating BCL-2 dependence [164]. Despite heavy reliance of MM on MCL-1 [165,166] and correlation of MCL-1 levels with poor prognoses [40], specifically targeting BIM-BCL-2 interactions induces apoptosis even in MCL-1 dependent MM [164]. While MCL-1 binds and sequesters BIM, the presence of sufficient BIM bound to other anti-apoptotics such as BCL-2 when targeted can also induce apoptosis, the effectiveness of which is importantly dictated by the quantity and distribution of BIM among the anti-apoptotics [164].

BH3 mimetics are potent small molecules used to release BH3 activators bound to anti-apoptotics. Currently, venetoclax (ABT-199) is the most promising small molecule BCL-2 antagonist. MCL-1-selective antagonists are in clinical trial [167]. The requirement for MCL-1 in myocardial homeostasis may preclude targeting MCL-1 [168]. Also, targeting BCL-x_L_ by ABT-737 is limited by its deleterious effects on normal platelet viability [169]. Clinically, venetoclax as a monotherapy is highly efficacious in Chronic Lymphocytic Leukemia (CLL) (that is BCL-2 dependent) [170]; however, effective only in a minority contingent of MM [171], with a recent phase 1 study suggesting only selective efficacy within the 11;14 myeloma patients [172].

Several studies have shown that co-targeting mitochondrial metabolism and the ETC can synergize with BH3 mimetics. We have shown that inhibition of glutamine metabolic pathways by glutamine deprivation or glutamine antagonist 6-diazo-5-oxo-L-norleucine (DON), modulates BCL-2 dependence, reducing the apoptotic threshold by increasing BIM-BCL-2 binding and the primed state, consequently increasing sensitivity to BH3 mimetics [119]. We have identified that targeting glutamine metabolism increases the primed state of the cell by increasing BIM binding to BCL-2 even across MM cells that are MCL-1 dependent i.e. having BIM bound primarily to MCL-1 [119]. In another study, DON was also shown to enhance apoptosis in combination with BCL-2 family antagonist Navitoclax (ABT-263) in glutamine addicted neuroblastoma and Ewing’s sarcoma cells overexpressing Myc [173]. Inhibition of GLS1 by CB-839 was found to reduce OXPHOS and selectively reduce proliferation and induce apoptosis in AML cells [174]. Supplementation with α-ketoglutarate and introduction of a hyperactive glutaminase C mutant rescued the cells from apoptosis. This study further showed that glutaminolysis inhibition by CB-839 synergized with venetoclax in AML. A recent study investigating the mechanism of action of combination treatment with venetoclax and hypomethylating agent azacitidine in older *de novo* AML patients revealed disruption of the TCA cycle, inhibition of ETC Complex II culminating in attenuation of OXPHOS leading to sensitization to venetoclax [175]. The inhibition of Complex II upon treatment with azacitidine was mediated by reduction in glutathione levels resulting in reduction of activating glutathionylation of Succinate Dehydrogenase A (SDHA) subunit of Complex II and selectively targeted the leukemic stem cell (LSC) population [175]. Further, in another study, investigation of the metabolism of LSCs from de novo AML patients revealed that they are dependent upon amino acid metabolism for the TCA cycle, and treatment with venetoclax and azacitidine depleted their amino acid pools as well as downregulated amino acid transporters [176]. Interestingly, AML blasts and LSCs from relapsed patients were found to be less reliant on amino acid metabolism and were resistant to venetoclax and azacitidine by utilizing fatty acid metabolism to support their TCA cycle. This suggests that relapse patients could potentially benefit from therapies combining venetoclax and azacitidine with inhibitors of fatty acid metabolism. This work illustrates how cancer cells can rewire their metabolism and develop resistance to therapy. Understanding the metabolism of relapsed/refractory cancer can help design combination therapies to overcome the resistance. Previously, Samudia et al., 2010, had demonstrated that de novo fatty acid synthesis supported fatty acid oxidation in leukemic cell lines OCI-AML3 and MOLM13. Inhibition of fatty acid oxidation or fatty acid synthesis by etomoxir (carnitine palmitoyltransferase-1 inhibitor) or orlistat (fatty acid synthase/lipolysis inhibitor) respectively, had anti-proliferative effects [177]. Moreover, treatment with etomoxir or orlistat sensitized these cell lines to apoptosis induction with ABT-737 (Bcl-x_L_, Bcl-2, and Bcl-w antagonist) and etomoxir also improved the efficacy of ABT-737 in a human AML murine model. While the precise mechanism of sensitization was not elucidated, this work indicated that fatty acid metabolism can be targeted to sensitize AML cells to BH3 mimetics. Additionally, recent work from our group has unveiled the importance of ETC function in modulating sensitivity of MM to venetoclax. We have demonstrated that inhibition of succinate ubiquinone reductase (SQR) by small molecule inhibitor thenoyltrifluoroacetone (TTFA) sensitizes venetoclax-resistant MM cells (under review, Nature Communications) [123]. This sensitization is mediated by upregulation of ATF4 upon TTFA treatment and subsequent increase in BIM and NOXA.

**Figure 2:**
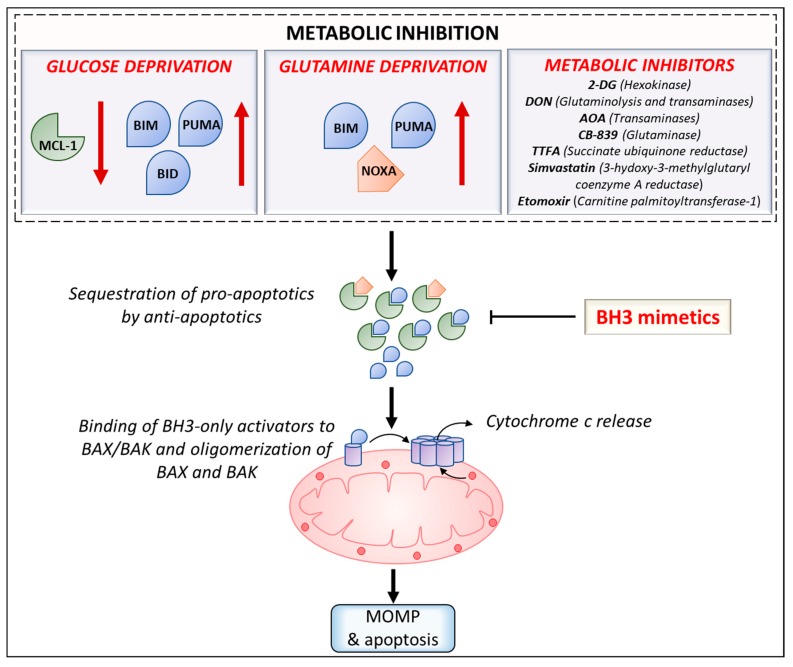
Metabolic regulation of BCL-2 proteins inducing sensitization to BCL-2 antagonists. Glucose/glutamine deprivation can modulate the levels of BCL-2 proteins resulting in changes in binding of BH3-only proteins with the anti-apoptotic proteins. Treatment with BH3-mimetics can release activators from anti-apoptotics. Unbound/released activators then activate BAX/BAK and initiate MOMP. A subset of metabolic inhibitors (pre-clinical leads and small molecules in clinical trials) that elevate the primed state and increase sensitivity to BCL-2-targeted BH3 mimetics have been shown along with their targets. Abbreviations: 2-DG: 2-deoxyglucose; DON: 6-diazo-5-oxo-L-norleucine; AOA: aminooxyacetic acid; TTFA: thenoyl trifluoracetone.

Lastly, efforts to repurpose FDA approved drugs for cancer treatment have led to investigation of efficacy of combining statins with BH3 mimetics. Several preclinical studies have shown that statins can act as anticancer agents as well as synergize with chemotherapeutics by enhancing cytotoxicity or by increasing cellular concentration of chemotherapeutics [178]. Inhibition of a key enzyme in the cholesterol synthesis pathway—3-hydoxy-3-methylglutaryl coenzyme A reductase (HMGCR)—by simvastatin was found to synergize with venetoclax in inducing apoptosis in diffuse large B cell lymphoma and AML [179]. HMGCR inhibition was shown to result in reduction in protein geranylgeranylation, subsequently leading to upregulation of PUMA and thus, priming the cancer cells for apoptosis. Furthermore, analysis of CLL clinical trials with venetoclax monotherapy revealed that statin users resulted in a higher rate of complete remission than patients without any background statin use. The mevalonate pathway has been shown to be upregulated in several cancers and its inhibition by statins and other inhibitors in combination with BH3 mimetics such as venetoclax can be an attractive therapeutic strategy for these malignancies [180]. Antibiotics constitute another promising group of compounds that can be repurposed for cancer therapy due to their inhibitory effects on mitochondrial function/metabolism. Ravà et al., 2018, reported that inhibition of mitochondrial translation by tigecycline sensitized MYC/BCL-2 double-hit lymphomas to venetoclax [181]. Related antibiotics doxycycline and tetracycline were also found to synergize with venetoclax. Further, tigecycline also synergized with venetoclax in treatment of mice engrafted with DHL cell lines or patient derived xenografts [181]. Recent work by Al-Zebeeby et al., 2019, on identifying metabolic inhibitors that can target resistance mechanisms in CLL has shown that inhibition of glutamine uptake and metabolism by various inhibitors (transaminase inhibitor amino-oxyacetic acid (AOA), ASCT2 inhibitor GPNA, glutaminase inhibitor CB-839, and glutamine: fructose-6-phosphate-amidotransferase (GFAT) inhibitor Azaserine) was found to sensitize BCL-x_L_ antagonist (A-1331852) resistant K562 cells to A-1331852 induced apoptosis [182]. Additionally, inhibition of downstream metabolic pathways such as reductive carboxylation, fatty acid synthesis and cholesterol synthesis by ATP-citrate lyase (ACLY) inhibitor SB20499, fatty acid synthase (FASN) inhibitor GSK2194069 and HMGCR inhibition by statins, respectively, sensitized resistant cells to A-1331852. Unpublished work from our group has also shown that AOA treatment sensitizes multiple myeloma cell lines to venetoclax. In sum, these studies demonstrate the benefit of developing co-targeting approaches using metabolic inhibitors and BH3 mimetics for cancer therapy. Various strategies to induce sensitization to BH3 mimetics by increasing the ‘primed’ state of cancer cells using metabolic inhibition are outlined in Figure 2.

## 7. Conclusions

Altered metabolism and the evasion of apoptosis are hallmarks of cancer cells. Targeting tumor metabolic dependencies is an attractive strategy for therapy development. However, the efficacy of monotherapy using metabolic inhibitors is limited by emergence of adaptive metabolic changes. Cancers cells are largely dependent upon pro-survival proteins of the BCL-2 family for evasion of apoptosis. Recent advances in understanding regulation of apoptosis have shown that BCL-2 proteins are under tight metabolic control. Several BH3 mimetics have been developed to target the primed state of cancer cells. Co-targeting approaches combining metabolic inhibitors, that increase the ‘primed’ state, and BH3 mimetics can be used to achieve potent drug synergies and further, expand the application of these individual therapies. Therefore, concomitant inhibition of the two hallmarks of cancer i.e. altered metabolism and the evasion of apoptosis is a promising strategy to effectively target various cancers.
